# Impact of Bystander Naloxone on Emergency Medical Transport Refusal After Opioid Overdose: A Statewide Retrospective Analysis

**DOI:** 10.5811/westjem.50754

**Published:** 2026-05-14

**Authors:** Daniella M. Carnevale, Peter Canning, Regina Kostyun, Richard Kamin

**Affiliations:** * University of Connecticut School of Medicine, Farmington, Connecticut; † University of Connecticut, John Dempsey Hospital, Department of Emergency Medicine, Farmington, Connecticut

## Abstract

**Introduction:**

The opioid epidemic remains a public health crisis in the United States. Naloxone is a cornerstone of overdose reversal, and its increasing availability to bystanders has improved immediate survival. However, little is known about how bystander naloxone administration influences use of emergency medical services (EMS), particularly patient refusal of transport. Understanding these dynamics is critical for development of EMS protocol and harm reduction strategies.

**Methods:**

We performed a retrospective cohort study of suspected opioid overdoses reported to the Connecticut Statewide Opioid Reporting Directive (SWORD) between November 1, 2019–June 30, 2024. The primary outcome was EMS transport refusal, defined as non-transport after naloxone administration. The primary exposure was initial naloxone administrator (bystander vs first responder). Secondary variables included naloxone dose frequency, patient demographics, and time. Bivariate tests compared group differences. We used multivariable logistic regression to assess the association between bystander naloxone and refusal, adjusting for covariates. To evaluate temporal trends, we performed separate logistic regression models with calendar quarter (Q) modeled as a continuous variable (Q1 2020–Q2 2024).

**Results:**

Among 15,025 nonfatal suspected overdoses involving naloxone in Connecticut, bystanders were initial administrators in 18%. Transport refusal occurred more often after bystander administration compared to first responder administration (16.1% vs 6.2%). In adjusted analyses, bystander administration was associated with nearly threefold higher odds of refusal (adjusted odds ratio [aOR] 2.90; 95% CI, 2.53–3.31). Multiple-dose incidents were associated with decreased refusal (aOR 0.83; 0.72–0.93). During the study period, bystander administration increased from 15% in Q4 2019 to 24% in Q2 2024, corresponding to a 3.8% increase in odds per quarter (OR 1.04; 95% CI 1.03–1.05, P < .001). Refusal more than doubled from 4% to 12%, with odds increasing 4.5% per quarter (OR 1.05; 1.04–1.06, P < .001).

**Conclusion:**

Bystander-administered naloxone is increasingly common and strongly associated with higher odds of EMS transport refusal. While refusal does not always equate to unsafe outcomes, it represents missed opportunities for initiation of medications for opioid use disorder, harm reduction counseling, and linkage to care. Emergency medical services agencies should consider strategies such as leave-behind naloxone, peer recovery coach deployment, and EMS-initiated buprenorphine to capitalize on these encounters.

## INTRODUCTION

The opioid epidemic remains a critical public health crisis in the United States, with opioid-involved overdoses rising at an alarming rate.^1^ In 2021 alone, opioid overdoses accounted for nearly 3 million years of life lost, about one in 22 deaths nationwide.^2^ In Connecticut, unintentional overdose deaths have risen by 306% over the past decade, with opioids implicated in 92% of cases in 2022.^3^ These events disrupt families, increase foster care placements,^4^ and impose a staggering economic burden of over $1 trillion annually.^5^–^6^

Naloxone, a high-affinity opioid antagonist first approved by the U.S. Food and Drug Administration in 1971, remains central to overdose reversal.^7^ Early efforts by harm reduction activists and the introduction of intranasal formulations expanded community access, empowering bystanders to intervene before first responders arrived.^8^–^10^ Evidence consistently demonstrates that bystander-administered naloxone is generally safe and effective in preventing immediate fatalities.^11^–^13^ Between 2020–2022, layperson naloxone use prior to emergency medical services (EMS) arrival increased by 43.5% nationally.^14^

While survival benefits of naloxone are clear, transport refusal following reversal has emerged as a challenge. Patients revived by naloxone, particularly by bystanders, may decline subsequent EMS transport. Motivations for refusal are complex, ranging from fear of withdrawal and stigma to prior negative healthcare experiences.^15^–^18^ Historically, transport was recommended for observation of recurrent toxicity, but emerging evidence supports selective treat-and-release strategies.^19^–^22^ Still, refusal may represent missed opportunities to initiate medications for opioid use disorder, provide harm reduction kits, or connect patients with peer recovery coaches. Localized data on transport refusal rates can help provide insight on current trends in different communities. Such data can then help inform potential outreach programs and both identify and address reasons for refusal.

In 2019, Connecticut launched the Statewide Opioid Reporting Directive (SWORD), a mandatory system capturing EMS-reported suspected opioid overdoses. The SWORD offers real-world, population-level insights to guide harm reduction and EMS practices.^23^ In this study we leveraged SWORD data to examine trends in bystander naloxone administration and EMS transport refusal from 2019–2024. A secondary aim assessed whether bystander administration was associated with higher refusal likelihood compared to first responder administration. We hypothesized that bystander-administered naloxone would be linked to increased odds of transport refusal among patients for whom 9-1-1 was activated.

## METHODS

### Study Design

This was a retrospective cohort study of suspected opioid overdoses in Connecticut reported in SWORD from November 1, 2019–June 30, 2024. Institutional review board approval was obtained (UConn Health IRB #25X-343-2). Methods adhered to recommended practices for medical record review studies as described by Worster and Bledsoe and colleagues.^24^

**Population Health Research Capsule**

What do we already know about this issue?

*Bystander naloxone use improves overdose survival, but its impact on EMS transport refusal and care linkage is not well defined*.

What was the research question?

*Is bystander naloxone administration associated with higher odds of EMS transport refusal after opioid overdose?*

What was the major finding of the study?

*Naloxone administration from a bystander was associated with 2.9-fold higher odds of transport refusal compared to first responders (aOR 2.90; 95% CI 2.53–3.31; P <.001)*.

How does this improve population health?

*EMS transport refusal after overdose reversal is an emerging concern, highlighting a growing disconnect between overdose reversal and potential engagement with post-reversal harm reduction*.

**Figure 1:**
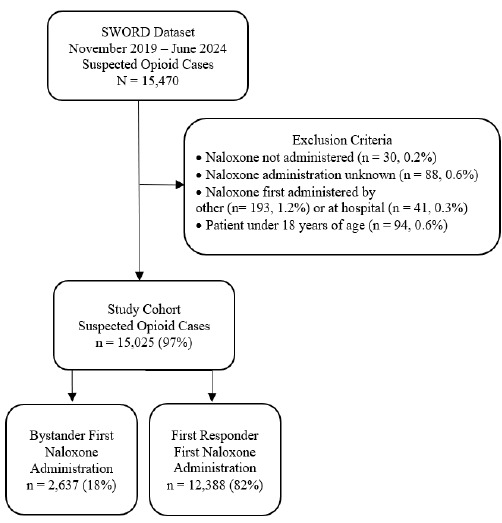
Flow diagram of study cohort selection from the Connecticut Statewide Opioid Reporting Directive database, November 2019–June 2024, in a study examining the odds of refusal of transport to hospital after naloxone administration by bystander vs first responder. *SWORD*, Connecticut Statewide Opioid Reporting Directive.

The SWORD database, managed by Connecticut’s Office of Emergency Medical Services and Poison Control Center at University of Connecticut Health, tracks opioid overdose cases in real time. Emergency medical services clinicians must report any 9-1-1 calls involving suspected opioid overdoses with reduced responsiveness or breathing problems. Reports are submitted via a dedicated electronic portal following case completion. Required fields include naloxone administration status, initial administrator identity, and transport disposition. Records were included if naloxone was administered for suspected opioid overdose in individuals ≥ 18 years of age (Figure 1).

Cases were excluded if they resulted in fatality, involved administration by “other” or “hospital,” or occurred in patients < 18 years of age (not eligible for independent refusal under Connecticut EMS protocols). Fatal cases were excluded because patients who did not survive to EMS evaluation could not undergo a transport refusal assessment and were, therefore, ineligible for the study’s primary outcome.

### Outcome and Associated Variables

The primary outcome was EMS transport disposition (transported vs refused transport). In Connecticut, there is no formal “treat-and-release” protocol and, thus, we used non-transport as a surrogate variable for patient refusal. The primary outcome was the identity of the initial naloxone administrator (bystander vs first responder). The “bystander” designation is determined by EMS personnel and includes anyone who administered naloxone prior to EMS arrival and is not affiliated with EMS, fire, or police. “Other” or “hospital” were excluded due to role ambiguity. Secondary outcomes included naloxone dose frequency (single vs multiple), age, sex, and time of year (calendar quarter [Q[).

### Statistical Analysis

Continuous variables are reported as means (standard deviation) if normally distributed and as medians (interquartile [IQR]) otherwise. Categorical variables are reported as counts (percentages). Chi-square tests assessed associations between initial naloxone administrator and categorical variables (sex, number of doses, and transport disposition). A Mann-Whitney U test compared age by administrator type due to non-normal distribution (Shapiro-Wilk test, *P* < .001). A multivariable logistic regression model evaluated the association between initial naloxone administrator and transport disposition, adjusting for age, sex, dose frequency, and calendar quarter (modeled continuously). Seasonality was not modeled separately, and calendar quarter was used to capture overall temporal trends. To assess temporal trends, separate logistic regression models were performed with transport refusal (yes/no) and bystander administration (yes/no) as dependent variables and calendar quarter as the independent variable. (We excluded Q4 2019 from trend models due to partial data capture).

We assessed model fit with the Hosmer–Lemeshow test. Adjusted odds ratios (aOR) with 95% confidence intervals were reported. We defined statistical significance as two-sided *P* < .05. Analyses were conducted using IBM SPSS v29.0 (International Business Machines Corporation, Armonk, NY). Cases with missing covariate data were excluded listwise in regression models. Age was missing in 261 cases (1.7%) and sex in 89 cases (0.6%).

## RESULTS

### Sample Characteristics

A total of 15,470 cases were initially identified. After exclusions, we included 15,025 nonfatal, suspected opioid overdoses involving naloxone administration in the final analysis. The study sample was 74% male (n = 11,035) and 26% female (n = 3,901), with 0.6% (n = 89) not reporting sex. The median age was 43 years (IQR 33–45). A total of 5,888 (39.2%) received a single dose of naloxone, and 9,137 (60.8%) received multiple doses. First responders administered the initial dose in 12,388 cases (82%), while bystanders administered it in 2,637 cases (18%). Among first responders, EMS administered the first dose of naloxone in 55% (n = 6, 846) of cases, fire services administered it in 31% (n = 3,798) of cases, and police administered it in 14% (n = 1,743) of cases.

### Trends in Bystander vs First Responder First Naloxone Administration

Table 1 summarizes patient characteristics of those who received the initial naloxone from a bystander and those who received it from a first responder.

Patients who were administered naloxone from a bystander were slightly younger (Mann-Whitney U = 14,007,545; Z = –9.156; *P* < .001), and multiple naloxone dose administrations were more common (χ^2^(df=1) = 434.93, *P* < .001).

### Bystander Naloxone Administration and Refusal

Overall, 1,196 patients (8.0%) refused transport after receiving naloxone. Among those who refused, 771 (64.5%) received naloxone from a first responder and 425 (35.5%) received naloxone from a bystander (χ^2^ (1) = 290.14, *P* < .001). Among refusals,836 patients (72%) were male and 324 (28%) were female. The median age was 42 (IQR 32–52). With regard to dosing, 471 (39%) received a single dose of naloxone and 61% received multiple doses. Table 2 provides the characteristics of patients who refused transport.

After adjusting for age, sex, dosage, and calendar quarter, bystander naloxone administration was associated with nearly threefold higher odds of refusal (Table 3). The model demonstrated good fit.

### Time Trends in Naloxone Administration and Refusal

**Figure 2:**
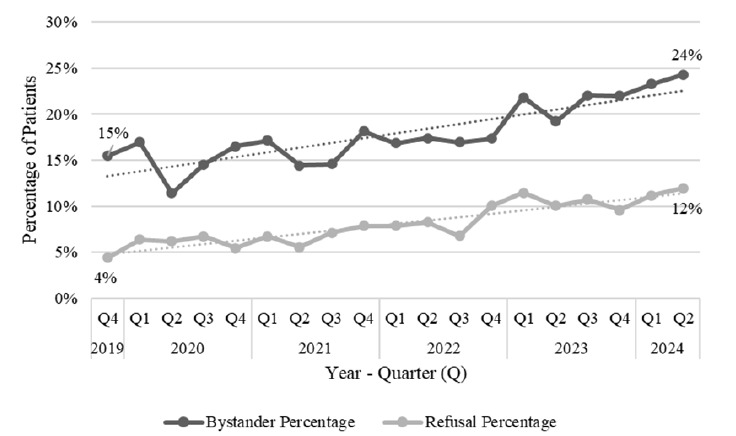
Trends in bystander-administered naloxone and EMS transport refusal among suspected opioid overdose patients, Connecticut SWORD database, Q4 2019–Q2 2024. Solid lines represent observed quarterly percentages; dotted lines indicate linear trend estimates. *EMS*, emergency medical services; *SWORD*, Statewide Opioid Reporting Directive.

As visualized in Figure 2, bystander-administered naloxone increased from 15% in Q4 2019 to 24% in Q2 2024, while transport refusals more than doubled during the same period, rising from 4.4% to 12%.

In separate time-trend logistic regression models, each quarter was associated with a 3.8% increase in the odds of bystander administration (odds ratio [OR] 1.04, 95% CI, 1.03–1.05, *P* < .001) and a 4.9% increase in the odds of refusal (OR 1.05, 1.04–1.06, *P* < .001). Both models demonstrated good fit by Hosmer-Lemeshow testing. These findings illustrate a steady upward trend in both bystander intervention and refusal of transport over time.

## DISCUSSION

A key strength of this study is the use of a mandatory, population-level statewide EMS dataset, which is rare in overdose epidemiology and enhances both the generalizability of results and their applicability to EMS and public health policy. In this statewide analysis of more than 15,000 suspected opioid overdoses, patients receiving naloxone from bystanders were nearly three times more likely to refuse EMS transport than those first treated by first responders. Both bystander naloxone administration and refusal rates increased over time, highlighting a shifting landscape of overdose response in Connecticut. Multiple doses were more common with bystander administration. Such higher rates of multiple-dose naloxone administration by bystanders may reflect untrained escalation or uncertainty in community response. The administration of multiple naloxone doses was associated with a lower likelihood of transport refusal. Lastly, older age of patient slightly reduced likelihood of transport refusal.

### Transport Refusal After Bystander Naloxone Administration

While research remains limited, prior studies suggest that physiologic, social, and contextual factors contribute to higher refusal rates when a bystander administers naloxone. People who use drugs have cited intolerable withdrawal, stigma, and expectations of poor hospital care as common reasons for declining transport.^17^ Higher rates were also observed during the COVID-19 pandemic, potentially due to fear of infection and strained systems.^25^ Additional associations include female sex, urban setting, and adverse social determinants of health, whereas family presence reduced refusal.^16^ In summary, prior studies have indicated that refusal is likely driven by a combination of individual and systemic factors. Future large-scale studies are needed to delineate the relative contributions of these factors, and findings should inform the refinement of public health policies and EMS practices to most effectively address them.

### Transport Refusal and Multiple Naloxone Doses

We found that patients receiving multiple naloxone doses were about 18% less likely to refuse transport. This pattern may reflect more severe or polysubstance overdoses, with residual sedation, confusion, or respiratory compromise. We also observed that bystanders were more likely to administer multiple doses of naloxone. On the other hand, EMS dosing is typically titrated to restore ventilation while minimizing withdrawal.^26^ Taken together, these findings suggest that both overdose severity and the identity of the administrator may interact with transport decisions in complex ways.

### Substances Involved and Contextual Considerations

Naloxone use patterns should be interpreted within the contemporary polysubstance overdose landscape. Many opioid overdoses during this period involve high-potency synthetic opioids such as fentanyl, often in combination with non-opioid substances (eg, stimulants or sedatives), which may increase overdose severity and complicate clinical response. However, community-based naloxone programs have continued to demonstrate effectiveness across these evolving drug eras.^27^ Toxicological testing of fatal overdoses in Connecticut from 2020–2023 showed that a large majority of opioid deaths involved fentanyl, and almost half of fentanyl overdoses co-involved cocaine. The animal tranquilizer xylazine was present in approximately one-fifth of overdose fatalities.^28^

### Recommendations for EMS Practice

Bystander naloxone programs save lives,^12^ but our findings suggest they may also increase the likelihood of refusal. Refusal is not always unsafe,^19^–^22^ but it reduces opportunities for initiation of medications for opioid use disorder and harm reduction engagement. For EMS clinicians, these encounters are, therefore, critical touchpoints. Even when transport is declined, EMS crews can deliver meaningful interventions such as leave-behind naloxone kits,^29^ peer recovery coach referrals,^30^ and initiation of buprenorphine when indicated.^31^–^35^ Leave-behind naloxone programs in Missouri,^36^ quick response teams in West Virginia,^37^ and integrated EMS-public health models in Ohio^38^ show how refusals can be reframed as outreach opportunities rather than dead ends. Furthermore, EMS-initiated buprenorphine has been shown to treat acute withdrawal with low risk of adverse events.

To help reduce refusals, EMS systems should emphasize training in compassionate communication that fosters trust. Future research should prioritize prospective studies and local data collection to clarify the drivers of refusal and assess the effectiveness of EMS-led interventions in closing the care gaps created by transport refusals. Particular attention should be given to social determinants, stigma, and geography as these may shape both refusal patterns and access to follow-up care.

While the field-based interventions show promise, their implementation requires consideration of feasibility and local context. Many of these strategies leverage existing EMS encounters and can be implemented with modest incremental cost, particularly when supported through public health or state-level funding mechanisms.^27^,^34^ Training requirements are variable but generally align with continuing education already provided to EMS clinicians, including overdose recognition, withdrawal assessment, and trauma-informed communication.^26^,^31^ Successful implementation often depends on partnerships between EMS agencies, public health departments, and community harm reduction organizations to facilitate referral pathways, medication access, and follow-up care.^28^,^35^,^36^ Tailoring these approaches to local resources and workforce capacity may help maximize impact while minimizing operational burden.

In summary, bystander naloxone administration is increasingly common and associated with higher EMS transport refusal. For EMS clinicians, refusals should not be seen as the end of care but as opportunities to connect patients with harm reduction and treatment resources. Expanding field-based interventions may ensure that overdose reversal serves as a gateway to recovery rather than an endpoint.

## LIMITATIONS

This study has several limitations. First, data are self-reported by EMS clinicians in the field and may be subject to human error or misclassification. All included cases were based on suspected opioid overdose at the time of naloxone administration and were not toxicologically confirmed. In some cases, bystander-administered naloxone may be administered in ambiguous clinical situations where opioid involvement is uncertain, particularly in community settings, which could influence patient response and subsequent transport refusal patterns. The database captures neither patients’ reasons for refusing transport nor long-term clinical outcomes, limiting our ability to assess downstream health impacts. Moreover, all non-transport cases were categorized as “refusals.” In Connecticut, EMS protocols require assessment of patient decision-making capacity prior to honoring refusal of transport, typically including evaluation of orientation, understanding of risks, and ability to communicate a choice. However, field conditions and documentation practices may still contribute to variability in recorded non-transport outcomes. We use the term “refusal” descriptively, without pejorative intent, to reflect operational EMS documentation rather than to imply judgment.

Additional limitations include incomplete data capture, especially during initial implementation (approximately 70% compliance in the first year), potentially underestimating early cases. Missing data were present for age (1.7%) and sex (0.6%). These cases were excluded listwise from regression models. Given the small proportion, this is unlikely to have materially influenced results. The dataset also excludes incidents where EMS was never contacted following bystander naloxone administration, limiting generalizability to community-managed overdoses. The sample was predominantly male; additionally, potential sex-based differences in transport refusal remain underexplored. Caution is warranted when generalizing findings to more diverse and/or nonbinary populations. Contextual factors like the COVID-19 pandemic may also have influenced patterns of administration and refusal. Because seasonality was not explicitly modeled, unmeasured seasonal variation and pandemic-related fluctuations may have influenced observed overdose and transport refusal trends. Finally, the chart abstractors were not blinded to the study hypothesis.

## CONCLUSION

In this statewide retrospective study, patients who received naloxone from bystanders were at significantly greater odds of refusing subsequent EMS transport compared to those treated first by first responders, and both bystander administration and refusals increased over the study period. These encounters represent important opportunities for EMS clinicians to offer harm resources and linkage to treatment, even when transport is declined. Strengthening field-based efforts may help ensure that overdose reversal serves as a gateway to recovery rather than an endpoint, ultimately improving patient-centered outcomes and reducing future harms.

## Figures and Tables

**Table 1:** Baseline characteristics of patients with suspected opioid overdose treated with bystander- vs first responder–administered naloxone in Connecticut, 2019–2024.

Characteristic	Bystander Group (n = 2,637)	First Responder Group (n = 12,387)	*P* value
Sex			.48
Male	1,915 (73%)	9,120 (74%)	
Female	705 (27%)	3,196 (26%)	
Age, median (IQR)	39 (31–51)	42 (33–55)	.001
Number of naloxone doses			.001
Single	559 (21%)	5,329 (43%)	
Multiple	2,079 (79%)	7,058 (57%)	
Refused transport	425 (16%)	771 (6.2%)	.001

Note: Age presented as median (interquartile range).

Missing data: from bystander group, 38 were missing age and 18 sex; and from first responder group, 223 were missing age and 71 sex.

**Table 2:** Characteristics of patients refusing transport following naloxone administration, stratified by initial naloxone administrator, in a study that found overdose victims receiving naloxone from bystanders were nearly three times more likely to refuse EMS transport than those receiving it from first responders.

Characteristic	Refused Transport (n = 1,196)	

	Bystander Group (n = 425)	First Responder Group (n = 771)
Sex		
Male	299 (70%)	564 (73%)
Female	122 (29%)	202 (26%)
Age, median (IQR)	40 (31–50)	43 (33–53)
Number of naloxone doses		
Single	114 (27%)	357 (46%)
Multiple	311 (73%)	414 (54%)

Values are presented as n (%) unless otherwise indicated. Age is reported as median (IQR).

*IQR*, interquartile range.

**Table 3:** Multivariable logistic regression of factors associated with emergency medical services transport refusal after naloxone administration in suspected opioid overdose patients, Connecticut SWORD database, 2019–2024.

	Adjusted Odds Ratio (95% CI)	*P* value
First naloxone administrator		
First responder	Reference	-
Bystander	2.90 (2.53–3.31)	<.001
Dosages		
Single	Reference	-
Multiple	0.83 (0.72–0.93)	.003
Age, per year	0.992 (0.987–0.997)	< .001
Sex		
Male	Reference	-
Female	0.96 (0.84–1.10)	.56
Time, per calendar quarter	1.05 (1.032–1.058)	< .001

*SWORD*, Statewide Opioid Reporting Directive.
